# Exploring the MAPPING application to facilitate risk communication and shared decision-making between physicians and patients with gynaecological cancer

**DOI:** 10.1136/bmjoq-2024-002776

**Published:** 2024-08-19

**Authors:** Mijra Koning, Christianne Lok, Dirk T Ubbink, Johanna Wilhelmina Maria Aarts

**Affiliations:** 1 Amsterdam UMC Locatie AMC, Amsterdam, Noord-Holland, The Netherlands; 2 Centre for Gynecological Oncology Amsterdam (CGOA), Amsterdam, The Netherlands; 3 NKI, Amsterdam, Noord-Holland, The Netherlands; 4 Department of Obstetrics & Gynaecology, Amsterdam UMC Location VUmc, Amsterdam, Noord-Holland, The Netherlands

**Keywords:** Shared decision making, Obstetrics and gynecology, Decision support, clinical

## Abstract

This is an observational study in which we evaluated current levels of risk communication (RC) among gynaecological oncologists and their view on the Mapping All Patient Probabilities in Numerical Graphs (MAPPING) application as a possible tool to facilitate RC and shared decision-making (SDM). In part A, we audio-recorded 29 conversations between gynaecological oncologists and patients when discussing treatment options. In part B, interviews were performed with eight gynaecological oncologists.

RC and SDM were measured using two observer-based measures, that is, the RC content (RCC) tool (scale 0–2) and the OPTION-5 instrument (scale 0–100). We used CollaboRATE questionnaire (scale 0–10) and a self-developed survey to assess patient-reported RC and SDM. In part B, we evaluated physicians’ attitudes regarding the use of the MAPPING application to support RC. Patients were minimally involved in the decision-making process (OPTION-5 25.9%±13.4 RCC 0.21±0.18). Patient-reported SDM was high (mean collaboRATE score 9.19±1.79) and patients preferred receiving numeric information, whereas most physicians used qualitative risk terms rather than exact numbers. In part B, gynaecologists had a positive attitude towards the MAPPING application. However, they stated that the app was difficult to use improvement of layout and better implementations are needed.

**WHAT IS ALREADY KNOWN ON THIS TOPIC**

- Risk communication is an important aspect of shared decision-making and stands as an essential element for ensuring well-informed consent from patients. However, the execution of risk communication poses a challenge for numerous healthcare professionals. Risk communication among gynaecological oncologists has not been previously studied and let alone how it could be improved.

**WHAT THIS STUDY ADDS**

- This study highlights the necessity for improvement in risk communication among gynaecological oncologists. The use of a visualisation tool, such as the Mapping All Patient Probabilities in Numerical Graphs (MAPPING) application, should be further explored as a potential facilitator to improve the communication of risks between patients and their doctors. However, physicians encountered several barriers to use and implement this tool in their outpatient clinic. Adaptations within the application are required to effectively integrate it into daily care.

**HOW THIS STUDY MIGHT AFFECT RESEARCH, PRACTICE OR POLICY**

- This study demonstrates that even nowadays risk communication remains challenging. Visualisation tools such as the MAPPING application could contribute to improved risk communication and enhanced shared decision-making. Future research should focus on implementing such tools and the impact on risk communication.

## Introduction

 Shared decision-making (SDM) is an important principle in modern healthcare.^1^ SDM improves quality of care and leads to higher patient satisfaction. Especially in cancer treatment, every treatment option comes with its own benefits and risks. Survival is often considered to be the most important outcome for patients with cancer. However, patients may weigh treatment options and outcomes very differently. Previous studies among patients with cancer have shown that they are sometimes willing to accept a higher risk of recurrence if that would decrease the risk of side effects and complications.^2 3^ This has also been investigated among women with gynaecological malignancies, such as endometrial or vulvar cancer.^3 4^

An important component of SDM is risk communication (RC). RC occurs when physicians explain benefits and risks of different treatment options to their patients. Patients need to receive understandable information about the advantages and disadvantages of a treatment option in order to make a decision that fits their preference and situation. However, RC is challenging, because both patients and doctors can have difficulties in understanding statistical information.^5^,^8^ Clinicians often underestimate low health literacy among their patients, which is present in 36% of the population.^9^ Health literacy is important for patients in order to engage in SDM^10^ and low statistical literacy is associated with adverse health outcomes.^6 11 12^

To support and improve RC, the visualisation of risks using graphical formats has been increasingly used. This appears to be a more effective way of understanding, processing and interpreting numerical information than when merely numerical information is presented.^813^,^15^ Limited research is available whether visualisation of risk improves RC and if this could lead to more SDM. We do know from previous studies with decision aids for patients with breast cancer that such tools can assist in the RC process.^16 17^ But visualisation of risks has never been investigated before in a gynaecological cancer population.

The Mapping All Patient Probabilities in Numerical Graphs (MAPPING) application has been developed to visualise the opportunities and risks of different treatment options for several illnesses through bar charts, icon arrays and natural frequency trees^18^ (www.mapping.nu). It is hypothesised that by improving the patients’ knowledge about the risks of possible treatment options, they will be better informed and better able to share their preferences regarding treatment.

As there is, to date, no previous study that assessed RC in gynaecological oncological practice, we first evaluated, in this study, the current level of RC as part of SDM in consultations between gynaecological oncologists and their patients. Second, we evaluated views and attitudes towards the MAPPING application as a possible tool to improve RC.

## Methods

### Setting and ethics

Annually, around 4500 women are diagnosed and treated for gynaecological cancer. In the Netherlands, gynaecological oncological care is centralised and provided in nine oncological centres. Patients diagnosed with gynaecological cancer are referred to one of these centres for their treatment. This study took place in two gynaecological oncological centres, together called the Center of Gynaecological Oncology Amsterdam. Together they provide care to almost 25% of the Dutch population (ie, 1000 patients). In total, 13 gynaecological oncologists work at these two hospitals. The medical ethics review board of both locations waived the need for a full review.

### Study design

This is an observational study consisting out of two parts, evaluating RC in current practice (part A) and clinicians’ attitudes towards the MAPPING application (part B) before its implementation in daily practice as a possible supporting tool for RC in the future.

### Part A

In this part, we aimed at evaluating the level of RC and SDM in conversations between patients and gynaecological oncologists in current practice. We performed both an observer-reported and patient-reported evaluation.

### Participants

We included 29 newly referred patients (age >18) with stage III or IV ovarian cancer, low, intermediate or high-risk endometrial cancer, or vulvar cancer. For all these patients, there were two or more treatment options to discuss during the first consultation. An overview of the treatment options is presented in online supplemental appendix 1. Patients without a good understanding of the Dutch language were excluded.

### Data collection

Eleven out of 13 gynaecological oncologists agreed to participate. Eligible patients were selected from the consultation schedules of these gynaecologists. One of the researchers informed the patient before the consultation about the study and asked informed consent. The gynaecologist was asked to audio-record the entire consultation. After the consultation, patients received a short questionnaire (online supplemental appendix 5).

### Outcome measures from the observers’ perspective

The audio-recordings were assessed by two researchers independently to score the level of patient involvement in the decision-making process using the Observer OPTION-5, and RC using the RC content (RCC) score.

### RC using RCC

RC was scored using the RCC.^19 20^ The RCC is an observational measure of clinical RC. The nine items can be rated on a 3-point Likert scale.^19^ Before using the RCC for assessment a few steps were followed to increase reliable use in a Dutch setting. First, we translated the RCC into Dutch for a better understanding, using the forward–backward translation.^21^ Second, we contacted the researcher who developed the tool for a better understanding of how to score the RCC. Third, we contacted the Amsterdam UMC RC group, experienced in using the RCC, to learn how to interpret the RCC items. The researcher was trained in scoring RC with the RCC one-on-one with a professional within this RC group, and this training was repeated after coding a few trial consultations. Then, we wrote a short manual in Dutch on how to score the RCC. The RCC score was computed by averaging responses to the nine questions (each question was scored 0–2).

### Level of SDM using Observer OPTION-5

The Observer OPTION-5 (‘observing patient involvement’) is a validated objective measurement instrument to measure the extent in which the physician involves the patient in the decision-making process. It consists of five items. Each item is scored from 0 (no effort made) to 4 (exemplary effort made).^22 23^ The total OPTION-5 score could range between 0 and 20.^23^ This was rescaled to a percentage of the maximum score. To minimise interobserver variability, two researchers scored these recordings independently. The results were compared and any discrepancies were discussed and resolved. We calculated a kappa value to assess interobserver agreement. When the lower 95% confidence limit was >0.6, the remaining conversations were scored by a single researcher.

### Outcome measures from the patient perspective

Patients were asked to participate in a short survey immediately after the consultation. In this survey also information about demographics, education level and diagnosis was collected. The questionnaire contained questions to gauge the patient’s recollection of the received information including risks, side effects, and treatment options. Additionally, we asked patients about preferences for RC. Furthermore, we included the CollaboRATE questionnaire,^24^ which is a brief tool to measure the level of SDM from the patient’s perspective.^25^ The tool consists of three questions about the patient’s encounter with their doctor. Patients can rate how much effort was made on three aspects of SDM using a ten-point Likert scale ranging from ‘no effort was made (0)’ to ‘every effort was made^10^ ’. Total scores were presented as a percentage of the maximum score. A mean and a top score were calculated.^24 25^

### Patient sample size

Our main outcomes were observer-reported RC, and observer-reported and patient-reported levels of SDM.^24 26^ Data analysis was conducted for the whole centre (Centre for Gynecological Oncology Amsterdam) instead of per physician. CollaboRATE items should be completed for a minimum of 25 clinical encounters in the group of interest to obtain reliable results.^25^ We took this as our minimally required number of participants.

### Data analysis

Baseline patient characteristics were analysed by using descriptive analysis, including means with SD and medians with IQRs if not normally distributed. All analyses were conducted using SPSS V.28 (IBM, Armonk, New York).

### Part B

In part B, eight gynaecological oncologists were invited for a semistructured interview to discuss the need for improvement of RC based on the results from part A. As a possible tool to support RC, the MAPPING application was demonstrated and the participating physicians were asked for their attitudes and views. All participating gynaecologists completed the Attitudes toward Decision aids fOr PatienTs (ADOPT) measure after the interview.

#### The MAPPING application

**Figure 1:**
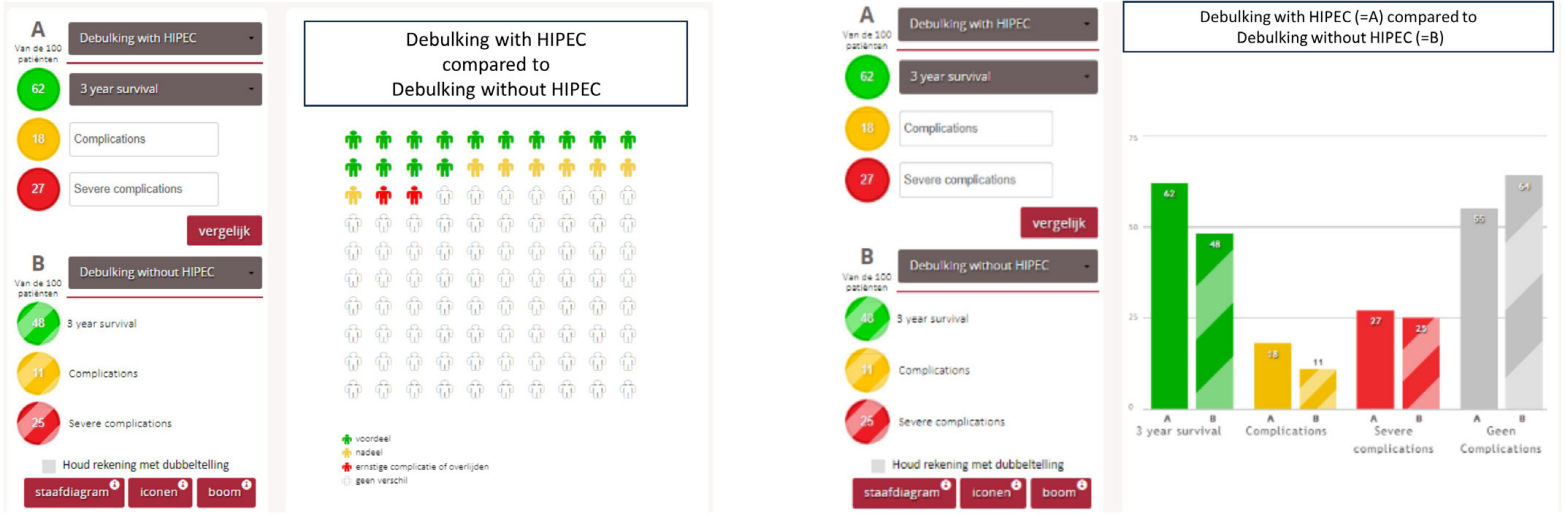
Bar charts and frequency trees in the Mapping All Patient Probabilities in Numerical Graphs (MAPPING) application, www.mapping.nu.HIPEC, Hyperthermic intraperitoneal chemotherapy.www.mapping.nu. HIPEC, Hyperthermic intraperitoneal chemotherapy.

The MAPPING application is a website that was developed to visualise the possible benefits and harmful effects, by default derived from (inter)national evidence-based guidelines of any treatment. For this part of the study, we entered risk data for the different treatment options for endometrial, ovarian and vulvar carcinomas. The application (www.mapping.nu) then could generate graphical displays of the data in the form of bar charts, figurine charts or natural frequency trees.^18^ These graphical displays were used during the consultation to explain the risks of various treatment options to the patients (see figure 1). Participants could select the visual display they preferred during the consultation.

### Data collection and analysis

Semistructured interviews were performed by one researcher following an interview guide (see online supplemental appendix 4). This interview guide was pilot tested. During the interview, the MAPPING application was shown and the interviewee was encouraged to think out loud while providing feedback. Each physician was interviewed once by the researcher, the researcher did not know the physicians on forehand. All interviews were recorded and transcribed verbatim. All transcripts were analysed using thematic analysis. One researcher (MK) coded all interviews, generating a list of initial codes. A second researcher (JWMA) used this list to independently code the same interviews. Both researchers then discussed their findings until consensus was reached. Overarching themes were derived from the codes. In addition, attitudes towards the MAPPING application were assessed using the ADOPT measure.^27^

The ADOPT measure is a tool to assess the clinician’s behaviour towards patient decision aids (PDAs) by using positive or negative adjectives. ADOPT consists of five positive adjectives (eg, easy) and five negative adjectives (eg, laborious) presented in a particular order. After the interview, gynaecologists were asked to select any words that described the use of the application as a PDA. They could select as many as they would like, as is instructed in the ADOPT validation paper. To identify whether clinicians had a positive or a negative attitude towards a PDAs, such as the MAPPING application, we calculated the number of positive and negative adjectives selected by the clinicians.

### Patient and public involvement statement

No patients or public were involved in the study design. However, patients were involved in the former development process of the MAPPING application.^16^

## Results

### Part A

Eleven gynaecological oncologists participated and each recorded on average three consultations. Between July 2021 and March 2022, 29 out of the 37 eligible patients who visited the outpatient clinics were included in this study (see online supplemental appendix 2 for a flowchart). Eight patients were excluded: six patients did not want to participate, one patient did not understand the Dutch language and one patient could not be reached for informed consent before the consultation. Two consultations failed to be recorded.

### Baseline patient characteristics

The patients’ mean age was 67.7±11.9 years. Sixteen were diagnosed with advanced stage ovarian cancer, six had endometrial cancer and seven had vulvar cancer. Most had a lower level of education (71.9%). Patient characteristics are shown in table 1.

**Table 1:** Baseline patient characteristics

	Current practice group (N=29)
Age (years; mean, SD)	67.7 (11.9)
Diagnosis (n, %)	
Ovarian cancer	16 (55,2)
Endometrial cancer	6 (20.7)
Vulvar cancer	7 (24.1)
Education level (n, %)	
No education or primary school	2 (6.9)
High school (low-medium level)	19 (65.5)
High school (high level)	1 (3.4)
Intermediate professional	2 (6.9)
Higher professional or university	4 (13.8)
Country of birth (n, %)	
Dutch	25 (86,2)
Non-Dutch*	4 (13.8)

* People from Iraqi, Chinese, Indonesian descent who understood the Dutch language sufficiently.

### Observer’s perspective

#### RCC scores

Mean RCC score was 0.21±0.18 on a scale from 0 to 2. RC was mostly performed verbally, by using terms such as ‘often’, ‘sometimes’ or ‘higher risk’. RCC item 7 occurred most frequently; in 65.5% of consultations. Means and frequencies of each item are shown in table 2.

**Table 2:** Mean scores per RCC item and frequencies

RCC item	(Mean, SD)	Frequencies (N, %)
1. Discussed the quality/strength/weakness (eg, validity, reliability, credibility) of the risk evidence	0	0
2. Specified the reference class (patient population) for whom the risk estimates apply	0.15 (0.53)	2 (6.9)
3. Specified the time period over which the risk estimates apply	0	0
4. Explained the magnitude of risk using both negative and positive frames	0.07 (0.38)	1 (3.4)
5. Explained risk estimates using both proportions (eg, ‘9 out of 100’) and percentages (eg, 9%)	0.07 (0.38)	1 (3.4)
6. Discussed differences between baseline risk and modified risk in absolute terms (absolute risk reduction) or both absolute and relative terms	0.07 (0.38)	1 (3.4)
7. Acknowledged general uncertainty in all risk estimates using qualitative terms	1.41 (0.93)	19 (65.5)
8. Acknowledged uncertainty due to chance or randomness (inability to predict single events)	0.15 (0.53)	2 (6.9)
9. Placed the magnitude of risks in context by comparing to risks of other outcomes(eg, other diseases, treatments, familiar events	0	0

RCCrisk communication content

### Observer OPTION-5

**Figure 2:**
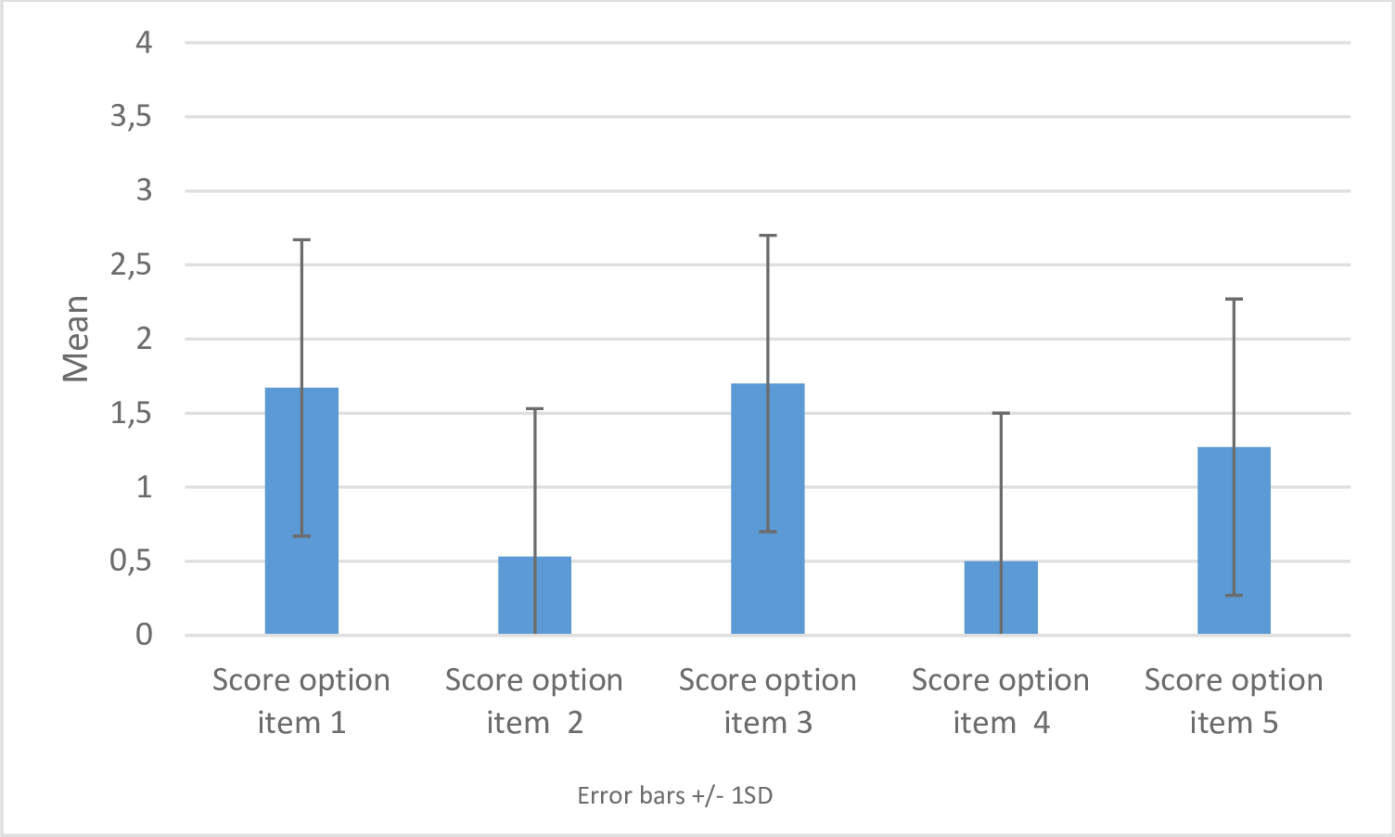
Mean SDM scores per OPTION-item. Mean SDM scores per OPTION-5 item are presented as bars. Error bars indicate one standard deviation. OPTION1: Justify the work of deliberation. ‘For the health issue being discussed, the physicians draw attention to or confirms that alternate treatment or management options exist or that the need for a decision exists’(1). OPTION2: Justify the work of deliberation as a team. ‘The physician reassures the patient or re-affirms that the clinician will support the patient to become informed or deliberate about the options(1)’ OPTION3: Inform, describe options, exchange view. OPTION4: Elicit patient preferences. OPTION5: Integrate preferences as the decision is made by the patient (1). 0 = No effort 1 = Minimal effort 2 = Moderate effort 3 = Skilled effort 4 = Exemplary effort (1).^23^

Mean OPTION-5 was low (25.9%±13.4). Gynaecologists scored best on OPTION-items 1 and 3 (see figure 2), with mean scores of 1.67±0.802 and 1.7±0.952, respectively. Lowest scores were found for OPTION items 2 and 4 with mean scores of 0.53±0.819 and 0.50±0.731, respectively (figure 2). Kappa value was 0.81 (95% CI 0.64 to 0.97).

### Patient’s perspective

#### CollaboRATE

Mean CollaboRATE-score was high: 9.19±1.79 (0–10). In 55.2% of the consultations a top score was given.

#### Survey

Twenty-eight out of 29 patients completed the survey. Gynaecologists mostly communicated risks verbally (55.2%). Some used drawings to explain risks (24.1%). In 31% of these consultations, numbers or percentages were provided. Of 93.1% of patients liked receiving information about risks. Patients preferred it the most when numeric information was communicated.

### Part B

#### Interviews with physicians

Eight gynaecological oncologists were interviewed. Mean interview duration was 15 min. Thematic analysis focused on facilitators and barriers for using the MAPPING application during the consultation. The codes that emerged from the thematic analysis were categorised according to a known framework in implementation studies^28^: factors related to the physician, the patient, the intervention and the context. An overview is presented in table 3.

**Table 3:** Overview of themes that are potential facilitators and barriers in the use of the MAPPING application

	**Facilitators**	Barriers
Physician-related factors		Physicians don’t know the specific numbers Physicians use verbal risk communication Personal preference Standard care/protocol
Patient-related factors	Patients want to be involved Patients must know the risks Risk information is personal	Patients do not want exact numbers Risk information is individual Patients do not want multiple choices in treatment options Patients only want to be cured Most patients are not able to handle risks/chances
Intervention-related factors	Illustrative Supportive Multiple display options User-friendly Useful Simple Evident	Numbers are generalising Cluttered Complicated Confusing Not intuitive
Context/societal-related factors	Obligatory in the context of the law	Time consuming

MAPPINGMapping All Patient Probabilities in Numerical Graphs

#### Patient-related factors

Differing patients’ preferences on how to receive risk information was mentioned as a barrier by several gynaecologists. ‘(*…) one patient wants to hear much more than the other.’ ‘Some patients say; I have no idea. Then I will not mention any studies, but there are also patients who say; well I have already searched and I have already seen two studies and with those patients I go deeper into it*.

A few stated that they think that patients do not want to hear exact numbers. *In my experience patients don’t want to hear exact numbers. On top of that it can be very difficult to name a number during the first consultation. Because this depends on the course of the treatment’*. On the contrary, one gynaecologist mentioned that the interpretation of risks is personal. *We (doctors) tend to decide whether a risk is big or small. This is quite personal, I might think 10% is a big risk but the patient might think otherwise. Another facilitator is that* that patients want to be involved in the decision making process. *Nowadays patients want to be more involved in the decision making process*.

#### Physician-related factors

Among the physician-related factors, there were only barriers in this study. Participants had a personal preference on how to communicate risk information to patients. Gynaecologists used verbal RC with words such as ‘sometimes’ and ‘often’ instead of numbers. They also experienced difficulties using the application because they often have one standardised treatment plan according to the guidelines, which they communicate with their patients. One gynaecologist stated that physicians have a strong personal preference as to the best treatment option: *But I don’t think there are multiple treatment choices to be discussed with the patient. Most of the time the most ideal option is clear-cut*. Another barrier among physicians was that they often do not know the exact numbers regarding treatment risks or survival.

#### Factors related to the MAPPING application

Seven out of eight liked the visualisation of risks in the MAPPING application. Facilitators were that the app is illustrative, evident and has multiple display options. Gynaecologists generally had a positive attitude towards the figurine chart. It was considered the best and most easy way to present and understand benefits and risks. Bar charts were easier to misinterpret, partly due to the marginal difference in colour between the bars.

Barriers on the other hand were that, in certain situations, the app was found confusing, for example, the use of both negative and positive outcomes in the same graph. *It might make it more insightful for some patients, but I am afraid it might lead to more white noise. (…) patients are not that highly educated*. Most found the application too complicated. *I am constantly looking at how to use it myself, let alone how it would be for a patient. For the average patient this will be a challenge (…)*. This was mainly due to the layout.

#### Context-related factors

The major barrier was the lack of time, as the application was not perceived time-efficient. Also, some participants did not consider the MAPPING application a better alternative for existing information tools, such as drawings on a notepad or the information on a known Dutch website. A facilitator in patient care context is that RC is obligatory in the context of the law, therefor using the MAPPING application ensures communication on risks.

### ADOPT measure: attitudes towards the MAPPING application

Five out of eight gynaecologists had a positive attitude. The majority of words chosen was positive (N=15). Words that were chosen most often were ‘collaborative’ and ‘effective’. See online supplemental appendix 3 for an overview of answers.

## Discussion

We investigated RC and SDM among gynaecological oncologists from an observer perspective and patient perspective. Improvements can be made regarding implementing RC into consultations. Most gynaecologists did not use numeric information when discussing the risks, but rather verbal terms or drawings to explain risks to patients. However, patients generally preferred numeric information. This could be improved by using a visual application, such as the MAPPING application. From the interviews, however, it was found that the MAPPING application in its current state was not implementation-ready.

RC showed room for improvement. This is particularly important as physicians are bound by law to explain the risks of possible side effects due to treatment options to their patients.^1^ To the best of our knowledge, no previous study investigated levels of RC in gynaecological cancer care. RC is, however, an important component of SDM. Without adequate RC, patients are not able to make a well-informed decision. This could imply that improvement of RC is needed to reach higher levels of SDM. A few PDAs to support women with gynaecological cancer in SDM have been developed. For instance, the use of PDAs with women with endometrial cancer led to more patient satisfaction. However, none of these studies focused on RC specifically.^29^ This is an important knowledge gap. In this study, patients expressed a preference for numerical information. However, the absence of such information during the consultation did not lead to a reduced patient satisfaction with the gynaecologists. A recent study of Richter *et al* showed that communicating risks is a process with multiple strategies and intertwined with clinical context. Some important factors are involved: the physicians’ experiences in explaining risk information, the way risk information is presented to the patient and the individual patient characteristics.^30^ There is evidence that when communicating risks, it is preferable to use numeric information and absolute terms,^31^,^33^ as was found in our study. Benefits of using numbers over words are that numbers are specific and ensure a better perception of risks than chance statements.^34^,^36^ Communicating risks in the form of descriptors rather than numbers leaves room for ambiguity.^32 37^ For example, the word ‘sometimes’ could mean 22% for one patient but 70% for another patient.^38^ Most importantly, patients themselves generally prefer numeric presentation of risks instead of verbal terms.^39 40^ Nevertheless, it remains difficult to use numeric RC in clinical practice due to low health literacy, innumeracy, statistical illiteracy or unawareness of available evidence.^541^,^43^ Also, numeric information for every patient-specific situation does not exist.

When showing the MAPPING application to patients, it is hypothesised that physicians automatically need to use numeric information and explain basic statistics such as absolute risk and risk reduction. The application enables to present risks in various graphical formats. Potentially this tool could thus improve RC. In a previous study, the MAPPING application was pilot tested among vascular surgeons who found the application simple and valuable.^18^ Unfortunately, we could not confirm these findings. As with every decision-making support tool, implementation in clinical practice is challenging. Moreover, research on the implementation of RC tools, such as the MAPPING application, is lacking. A recent study investigated how to perform personalised RC in patients with breast cancer, but this study did not focus on implementation.^44^ There are decision-making support tools such as decision aids, consultation cards and risk ladders^45^,^46^. These decision aids also inform patients of risks using a form of graphical displays.^47^ These interventions helped patients to be better informed as they obtained a greater perception of risks and were thus more equipped for the SDM process.^48^

The identification of barriers and facilitators for implementation of RC could help improve the MAPPING application and design an implementation strategy. These are in line with previous research. Facilitators have, for instance, positive effect on the consultation and patient outcomes.^49^ Future research should, therefore, focus on evaluating the effect of adequate RC and possible use of a supporting tool on the consultation and patient outcomes. This evidence could support easier implementation. One of the barriers is the fact that risk numbers in the tool not being applicable because of differing patient characteristics and clinical context.^49^ In future, implementation efforts should thus be stressed that numbers in the MAPPING application can be adjusted to every individual patient. To address gynaecologists’ concerns regarding lay out and use of the application, improvements to the application itself should be done in cocreation with them and patients. It is recommended to develop a training for physicians on RC and the use of the MAPPING application.

### Strength and limitations

This study has some strengths. First, the study was performed in two hospitals that treat approximately 25% of all Dutch gynaecological patients with cancer. Our study population could thus be considered a reliable representation. Second, we evaluated RC and SDM from two different perspectives. Third, we used validated measures. There are some limitations too. First, in some cases, another conversation with the radiation oncologists followed. In this conversation, more extensive RC likely took place, which was not recorded. Second, the validity of the RCC is not certain as it was not yet available in Dutch. However, we followed a structured translation protocol and consulted the researcher who developed it for adequate understanding of the RCC. Third, it is debatable whether the RCC is the right tool in this setting. When scoring the RCC, it is possible to score the items from the RCC with 1, for example, ‘erroneous information is given’. This does not seem to apply for physicians as it can be assumed specialists do not provide incorrect information to patients. Moreover, the RCC items are very specific and not applicable in all clinical settings. However, to the best of our knowledge, no other observational measures for RC are available. Another aspect we need to take in mind is that patients and physicians knew that the consultation was being recorded. This may influence the results since the gynaecologists might alter their approach, and the patient might focus on different aspects as a result.

## Conclusion

Observer-reported and patient-reported RC among gynaecological oncologists showed room for improvement. RC is usually performed using descriptive terms, while patients prefer numeric information. An RC tool such as the MAPPING application could aid physicians in using numeric RC with patients. However, the application first needs improvements and a structured implementation plan. Also physicians stated that there were several limitations, which complicated the usage of this tool in the consultation room. Further research is needed to evaluate if the MAPPING application could be implemented in daily care and if this would lead to better RC, possibly resulting in higher SDM levels.

## supplementary material

10.1136/bmjoq-2024-002776 online supplemental file 1

10.1136/bmjoq-2024-002776 online supplemental file 2

10.1136/bmjoq-2024-002776 online supplemental file 3

## Data Availability

Data are available upon reasonable request.
