# Supplementary material for: Exploring the MAPPING application to facilitate risk communication and shared decision-making between physicians and patients with gynaecological cancer
Source: BMJ Open Qual. 2024 Aug 19;13(3):e002776. doi: 10.1136/bmjoq-2024-002776 (PMC11337712; doi:10.1136/bmjoq-2024-002776)
Supplement: online supplemental file 1 [file bmjoq-13-3-s001.pdf]

APPENDICES

APPENDIX I. Treatment option per type of cancer

**Type of cancer**	**Stage high/low/medium risk**	**Treatment options**
Ovarian cancer	III	Cytoreductive surgery with or without HIPEC
	IV	Cytoreductive surgery or no surgery
Endometrial cancer	Intermediate or high-risk	Pelvic lymph node dissection, or adjuvant radiotherapy or no adjuvant treatment
	Low or intermediate risk	Vaginal brachytherapy or no adjuvant therapy
	Stage III	Chemo -and radiotherapy or radiotherapy alone
**Vulvar cancer**		Radical excision and sentinel node biopsy or groin lymph node dissection or adjuvant therapy

APPENDIX II. Flow chart of patients included in the study

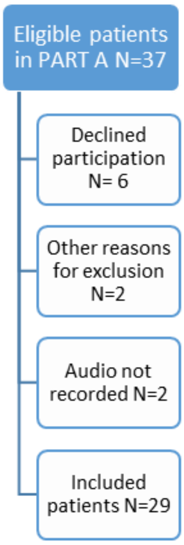

APPENDIX III *ADOPT words chosen by physicians*

	**Words**	**Frequencies (N,%)**	**Total neg/pos (N,%)**
**Positive**	Collaborative	5 (62,5)	15 (62,5)
	Time-saving	1 (12,5)	
	Easy	3 (37,5)	
	Effective	4 (50)	
	Necessary	2 (25)	
**Negative**	Unfamiliar	3 (37,5)	9 (37,5)
	Inefficient	1 (12,5)	
	Laborious	3 (37,5)	
	Inconvenient	1 (12,5)	
	Unrealistic	1 (12,5)	
