# Supplementary material for: Exploring the MAPPING application to facilitate risk communication and shared decision-making between physicians and patients with gynaecological cancer
Source: BMJ Open Qual. 2024 Aug 19;13(3):e002776. doi: 10.1136/bmjoq-2024-002776 (PMC11337712; doi:10.1136/bmjoq-2024-002776)
Supplement: online supplemental file 2 [file bmjoq-13-3-s002.pdf]

Appendix IV – Interview guides in Dutch and English translation

Interview Guide Mapping App Behandelaar (Dutch)

Om te kijken wat de opvattingen zijn onder onder de geïncludeerde behandelaren over Shared decision making en de invloed van de Mapping app op de risicocommunicatie, hebben we een interview guide gemaakt. Zo is het interview met elke behandelaar vergelijkbaar.

<u>Wat betekent samen beslissen voor u?</u>

- Hoe past u samen beslissen toe tijdens uw consult?
- Wat voor informatie geeft u de patiënt om mee te kunnen beslissen?
- Heeft de Mapping App effect op het samen beslissen, zo ja, hoe?

<u>Kunt u mij vertellen hoe u over risico's spreekt met patiënten?</u>

- Gebruikt u percentages of getallen, kunt u uitleggen waarom wel/niet?
- Gebruikt u plaatjes/tekeningen?
- Hoe heeft de Mapping App uw aanpak beïnvloed, zo ja, hoe dan?
- Heeft u het gevoel dat de Mapping App het begrip onder patiënten beïnvloed, zo ja, hoe?

<u>Wat is de algemene indruk van de Mapping App?</u>

- Wat vind u van de gebruiksvriendelijkheid?
- Wat vind u van de lay-out?
- Wat vind u van de gebruikte grafieken?

<u>Gaat u de Mapping app in de toekomst vaker gebruiken, zo ja/nee, waarom?</u>

- Zijn er specifieke aspecten die het gebruiksgemak bevorderen/verslechteren?
- Wat zou u veranderen/verbeteren?

English translation

What does shared decision-making mean to you?

- How do you implement shared decision-making during your consultations?
- What kind of information do you provide to the patient to enable them to participate in decision-making?
- Does the Mapping Application affect shared decision-making, and if so, how?

Can you tell me how you discuss risks with patients?

- Do you use percentages or numbers, and can you explain why you use them or why you don’t use them?
- Do you use pictures/drawings to explain risks?
- How has the Mapping App influenced your approach for discussing the risks?t
- Do you feel that the Mapping App affects patients' understanding, and if so, how?

What is your overall impression of the Mapping App?

- What do you think about its user-friendliness?
- What do you think about the layout?
- What do you think about the graphs the mapping application uses?

Will you use the Mapping App more frequently in the future, can you explain why you would or why you wouldn’t ?

- Are there specific aspects that improve/worsen user-friendliness ?
- What would you change/improve?
