# Supplementary material for: Exploring the MAPPING application to facilitate risk communication and shared decision-making between physicians and patients with gynaecological cancer
Source: BMJ Open Qual. 2024 Aug 19;13(3):e002776. doi: 10.1136/bmjoq-2024-002776 (PMC11337712; doi:10.1136/bmjoq-2024-002776)
Supplement: online supplemental file 3 [file bmjoq-13-3-s003.pdf]

APPENDIX V Questionnaire for patients

Date:
Doctor:
Interviewer:
Patient/Observation number:

Age:

Highest level of education completed:

❑ No education (primary education: not completed)

❑ Primary education (elementary school, special elementary education)

❑ Lower or preparatory vocational education (such as LTS, LEAO, LHNO, VMBO)

❑ Secondary general education (such as MAVO, (M)ULO, short MBO, VMBO-t)

❑ Secondary vocational education and apprenticeship education (such as long MBO, MTS, MEAO, BOL, BBL, INAS)

❑ Higher general and pre-university education (such as HAVO, VWO, Atheneum, Gymnasium, HBS, MMS)

❑ Higher vocational education (such as HBO, HTS, HEAO, HBO-V, candidate scientific education)

❑ Scientific education (university)

❑ Other, namely: . . . .

If you think back to your visit to your healthcare provider today...

How much effort was made to help you understand your health situation?

0 1 2 3 4 5 6 7 8 9

No effort All effort

How much effort was made to listen to the things that are important to you when it comes to your health situation?

0 1 2 3 4 5 6 7 8 9

No effort All effort

How much effort was made to take into account the things that are important to you when choosing the next step?

0 1 2 3 4 5 6 7 8 9

No effort All effort

Did the doctor explain to you if there are multiple treatment options available?

o Yes
o No
o I don't know

How many treatment options are available to you?

o 1
o 2
o 3
o 4
o 5

Did the doctor tell you about the risks of side effects or complications of the treatment options?
o Yes
o No
o I don't know

Can you give an example of the risks of the treatment that the doctor explained to you?

Did the doctor tell you about the risk of the disease recurring?

o Yes
o No
o I don't know

When discussing the risks, did the doctor mention specific numbers or percentages?
o Yes
o No
o I don't know

Do you remember the exact numbers that the doctor mentioned?
o Yes
o No
o Not applicable. The doctor did not mention any numbers

How did the doctor explain the numbers or risks to you?
o Orally
o Via the computer
o With a drawing
o With pictures
o Other, namely: . . . .

Did you find it helpful that the risks were discussed?
o Yes
o No

Did you find it helpful that specific numbers and/or percentages were mentioned?
o Yes
o No
o Not applicable. No specific numbers were mentioned

What did you think of the amount of information you received from the doctor?
o Too much information
o Too little information
o Just enough information

Do you feel that you were able to make a good decision about the treatment based on the information from the doctor?
o Yes
o No
o Not applicable. No decision has been made yet

Do you feel that you were able to choose the treatment yourself?
o Yes
o No
o Not applicable

What treatment options are available to you?
(You can select multiple answers)
o Only surgery
o Surgery + HIPEC (chemoperfusion)
o Surgery + radiation
o Surgery + chemotherapy
o Chemotherapy
o Radiation
o Chemotherapy + radiation

Which treatment carries the most risk of side effects or complications?
o Surgery
o Surgery + HIPEC (chemoperfusion)
o No treatment or surgery
o Chemotherapy
o Makes no difference
o Don't know
o No side effects were discussed
o Surgery + chemotherapy

Which treatment has the least chance of the disease recurring?
o Surgery
o Surgery + HIPEC (chemoperfusion)
o No treatment or surgery
o Chemotherapy
o Makes no difference
o Don't know
o This was not discussed
o Surgery + chemotherapy
o Not applicable

If the MAPPING application was used, would you like it to be used again in a future conversation when discussing risks?
o Yes
o No
